# Supplementary material for: Systemic Inflammation in Hip Fracture and Osteoarthritis: Insights into Pathways of Immunoporosis
Source: Int J Mol Sci. 2025 Sep 19;26(18):9138. doi: 10.3390/ijms26189138 (PMC12470133; doi:10.3390/ijms26189138)
Supplement: Supplementary file 1 [file ijms-26-09138-s001.zip › ijms-3869179-supplementary.pdf]

Table S1. Full Correlation matrix among inflammatory markers, FRAX scores, and BMD

	SII	P-VALUE	CRP	P-VALUE	FRAXT	P-VALUE	FRAXC	P-VALUE	CAD-BMD	P-VALUE	LUM-BMD	P-VALUE	WRIST-BMD	P-VALUE
**SII**	1.000	0.000	0.189	0.426	-0.015	0.951	-0.083	0.728	-0.188	0.428	-0.138	0.561	-0.178	0.452
**CRP**	0.189	0.426	1.000	0.000	0.076	0.750	0.039	0.871	-0.633	0.003	-0.600	0.005	-0.602	0.005
**FRAXT**	-0.015	0.951	0.076	0.750	1.000	0.000	0.911	2359150.979	-0.325	0.162	-0.200	0.398	-0.229	0.331
**FRAXC**	-0.083	0.728	0.039	0.871	0.911	2359150.979	1.000	0.000	-0.400	0.080	-0.310	0.183	-0.323	0.165
**CAD-BMD**	-0.188	0.428	-0.633	0.003	-0.325	0.162	-0.400	0.080	1.000	0.000	0.877	39455464.329	0.924	589169.133
**LUM-BMD**	-0.138	0.561	-0.600	0.005	-0.200	0.398	-0.310	0.183	0.877	39455464.329	1.000	0.000	0.960	1932.033
**WRIST-BMD**	-0.178	0.452	-0.602	0.005	-0.229	0.331	-0.323	0.165	0.924	589169.133	0.960	1932.033	1.000	0.000

Abbreviations: SII, systemic immune-inflammation index; CRP, C-reactive protein; FRAX-T, 10-year probability of a major osteoporotic fracture; FRAX-C, 10-year probability of hip fracture; CAD–BMD, total hip BMD; LUM–BMD, lumbar spine BMD; WRIST–BMD, distal radius BMD.
